# Identification of Barrett's esophagus in endoscopic images using deep learning

**DOI:** 10.1186/s12876-021-02055-2

**Published:** 2021-12-17

**Authors:** Wen Pan, Xujia Li, Weijia Wang, Linjing Zhou, Jiali Wu, Tao Ren, Chao Liu, Muhan Lv, Song Su, Yong Tang

**Affiliations:** 1 Department of Digestion, West China Hospital of Sichuan University, Chengdu, 610054 Sichuan China; 2 Department of Digestion, The Hospital of Chengdu Office of People’s Government of Tibetan Autonomous Region, Ximianqiao Street No.20, Chengdu, 610054 Sichuan China; 3 Department of General Surgery (Hepatobiliary Surgery), The Affiliated Hospital of Southwest Medical University, Taiping Street No.25, Luzhou, 646000 Sichuan China; 4 School of Information and Software Engineering, University of Electronic Science and Technology of China, 4 North Jianshe Road, Chengdu, 610054 Sichuan China; 5 Department of Anesthesiology, The Affiliated Hospital of Southwest Medical University, Taiping Street No.25, Luzhou, 646000 Sichuan China; 6 Department of Digestion, The Affiliated Hospital of Southwest Medical University, Taiping Street No.25, Luzhou, 646000 Sichuan China; 7 School of Computer Science and Engineering, University of Electronic Science and Technology of China, 4 North Jianshe Road, Chengdu, 610054 Sichuan China

**Keywords:** Barrett's esophagus, Esophagoscope, Deep learning, Fully convolutional networks, Segmentation

## Abstract

**Background:**

Development of a deep learning method to identify Barrett's esophagus (BE) scopes in endoscopic images.

**Methods:**

443 endoscopic images from 187 patients of BE were included in this study. The gastroesophageal junction (GEJ) and squamous-columnar junction (SCJ) of BE were manually annotated in endoscopic images by experts. Fully convolutional neural networks (FCN) were developed to automatically identify the BE scopes in endoscopic images. The networks were trained and evaluated in two separate image sets. The performance of segmentation was evaluated by intersection over union (IOU).

**Results:**

The deep learning method was proved to be satisfying in the automated identification of BE in endoscopic images. The values of the IOU were 0.56 (GEJ) and 0.82 (SCJ), respectively.

**Conclusions:**

Deep learning algorithm is promising with accuracies of concordance with manual human assessment in segmentation of the BE scope in endoscopic images. This automated recognition method helps clinicians to locate and recognize the scopes of BE in endoscopic examinations.

## Introduction

Barrett's esophagus (BE) is a precancerous state caused by damages to the inner lining of the squamous esophageal mucosa, characterized by a change of the normal stratified squamous epithelium lining esophagus to a metaplastic columnar epithelium with goblet cells [1]. BE is the only known histological precursor of esophageal adenocarcinoma (EAC) [2].]. It has been reported that EAC is associated with high mortality (5-year survival rate < 20%) and increasing incidences [3–6]. EAC patients with a prior diagnosis of BE normally have better outcomes than patients without a prior diagnosis of BE [7]. Therefore, early detection and appropriate treatment of BE are crucial for effective prevention of the development of EAC. At present, the most common screening method for BE is a pathological biopsy using samples obtained through esophagoscopy (ESO). However, due to the individual variations of the shapes, appearances, and textures of BE, accurate identification and location of the BE scope are still challenging. Moreover, the locating of BE relies on the individual experience of endoscopists, which might further introduce variations and bias. These problems might cause time consumption and misjudgments, probably led to delays in the identification or misdiagnosis of BE, and finally influenced the follow-up treatments. Therefore, to overcome these difficulties, it is necessary to further improve the efficiency and accuracy of BE identifying and locating under endoscopic examinations.

Recent years have witnessed tremendous development in artificial intelligence (AI), especially the emerging deep learning (DL) has achieved unprecedented successes in various domains with groundbreaking performance on par with human capabilities [8]. More recently, there is a trend of applying DL in healthcare and clinical applications [9, 10]. As a subbranch of AI, DL utilizes multiple layers of neurons to extract abstract patterns from data. In image analysis, DL shows encouraging potentials in tasks of segmentation, classification, and prediction [11–13]. A growing body of literature developed DL methods in analyses of medical images such as ultrasound, CT, MRI, X-ray [14–16].

Recently, DL has been gradually utilized in endoscopic image analysis of colon, stomach, and intestine, etc., with encouraging performance in identifying and diagnosing diseases such as tumors, polyps, and ulcers [17–19]. Meanwhile, several studies applied DL in the classification and segmentation of esophageal lesions [20–26]. However, there is still a lack of reports of developing DL methods dedicated to BE identification. Mendel et al. adopted a migration-based learning approach to segment endoscopic images included cancer and BE [20]. Wu et al. used a convolutional neural network (CNN) to segment endoscopic images of cancer, BE, and inflammation [21]. In a recently published study, a depth estimator network was used to measure C&M scores including the BEA [27]. Because of the importance of early diagnosis of BE in the prevention of EAC, it is worth further investigating DL in BE diagnosis with a sufficient BE sample size [28]. Previous study suggested that in Asia including China, short-segment Barrett’s esophagus was more common [29]. Moreover, for the cases of short-segment BE, it is relevantly easier to make accurate endoscopic diagnosis. Therefore, we focused on patients with BE less than 7 cm in this work.

The objective of this study was to develop a fully automated DL method for early-accurate segmentation and identification of BE in endoscopic images. We included 443 endoscopic images from 187 BE patients. The DL method could accurately identify and segment the scopes of BE, which could further facilitate the following endoscopic surveillance and treatment of BE.

## Materials and methods

**Fig. 1:**
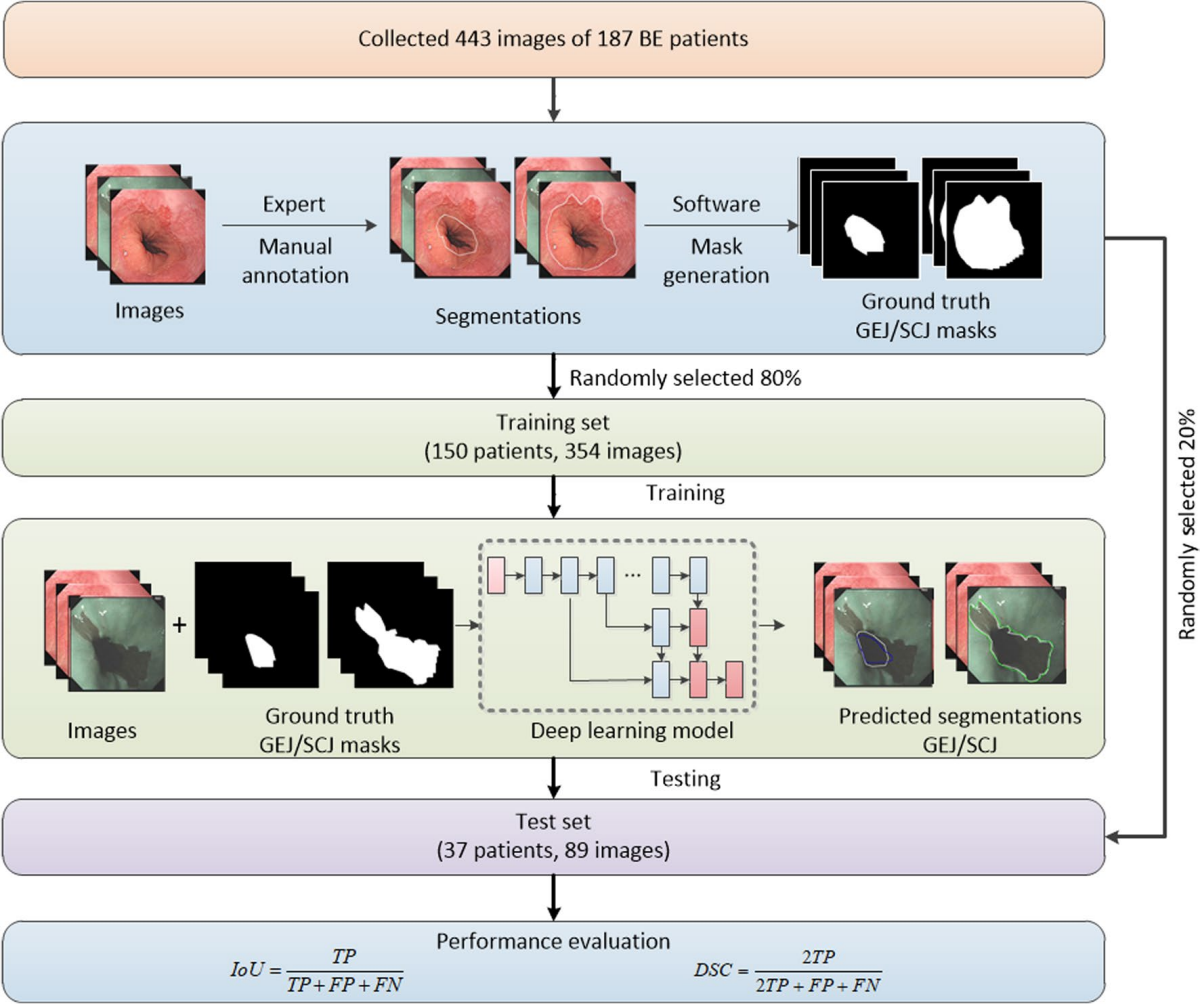
Overall workflow of this study

The overall workflow of this study was illustrated in Fig. 1. First, patients were included, and the endoscopic images were obtained. Next, the BE regions were annotated in the endoscopic images by experts. Based on the raw images and annotation information, the deep learning segmentation algorithms were trained and evaluated in training and validation datasets, respectively. Finally, the performance was summarized and reported.

### Patient characteristics

In our retrospective study, a total of 187 patients examined using endoscopy between January 2015 and June 2019 were included in the Hospital of Chengdu Office of People’s Government of Tibetan Autonomous Region. The BE conditions were confirmed by pathological examinations. All data were anonymized, and an Ethics Approval was granted by the Ethics Committee of Hospital of Chengdu Office of People’s Government of Tibetan Autonomous Region (No. 201920).

### Image acquisition

We obtained 443 endoscopic images from a total of 187 clinical cases (Table 1), and the instruments used in the examinations were Olympus GIF-HQ290, GIF-Q260 gastroscope (Olympus Company, Japan). The esophagus was cleaned and examined with white light, narrow band imaging, and staining endoscopy. The BE scope was recorded according to the Prague classification system. The endoscope was positioned proximally to the GEJ, and the endoscopic image was taken. Meanwhile, the biopsy samples were obtained using biopsy forceps, and the final diagnoses were proved by pathologists.

**Table 1:** Patient characteristics (training set and test set)

		Total	Training set	Test set	*p* value
Cases, n(%)		187 (100.00%)	150 (80.21%)	37( 19.79%)	
Sex	Male, n(%)	139 (74.33%)	110 (73.33%)	29 (78.28%)	.53
	Female, n(%)	48 (25.67%)	40 (26.67%)	8 (21.62%)	
Age, years, mean ± SD*		53.96 ± 10.56	54.15 ± 10.34	53.16 ± 11.56	.31
BMI, kg/m2, Median(IQR)**		23.67 (22.57–24.63)	23.70 (22.26–24.67)	23.60 (23.16–24.50)	.31
Barrett’s maximum length, cm	< 3	136 (72.73%)	108 (72.00%)	28 (75.68%)	.65
	≥ 3	51 (27.27%)	42 (28%)	9 (24.32%)	

^*^Age is expressed as mean ± SD (standard deviation)

^**^BMI, body-mass index; IQR, interquartile range

### Image annotation

To obtain the ground truth of the BE scopes in images, we invited two senior endoscopists with over 15 years’ experience to manually draw the outlines of the scopes using one in-house developed software. More specifically, the rims of the GEJ and SCJ were delineated to define the BE scopes. The experts were trained to follow the same quality standard before conducting the tasks. The first expert annotated all images, and the results were confirmed by the second expert. For any disagreement, the two experts discussed and made necessary new annotations until consensus was reached. The annotation information was later extracted to generate segmentations as ground truths for later DL algorithm training and evaluation.

### Deep learning algorithm

**Fig. 2:**
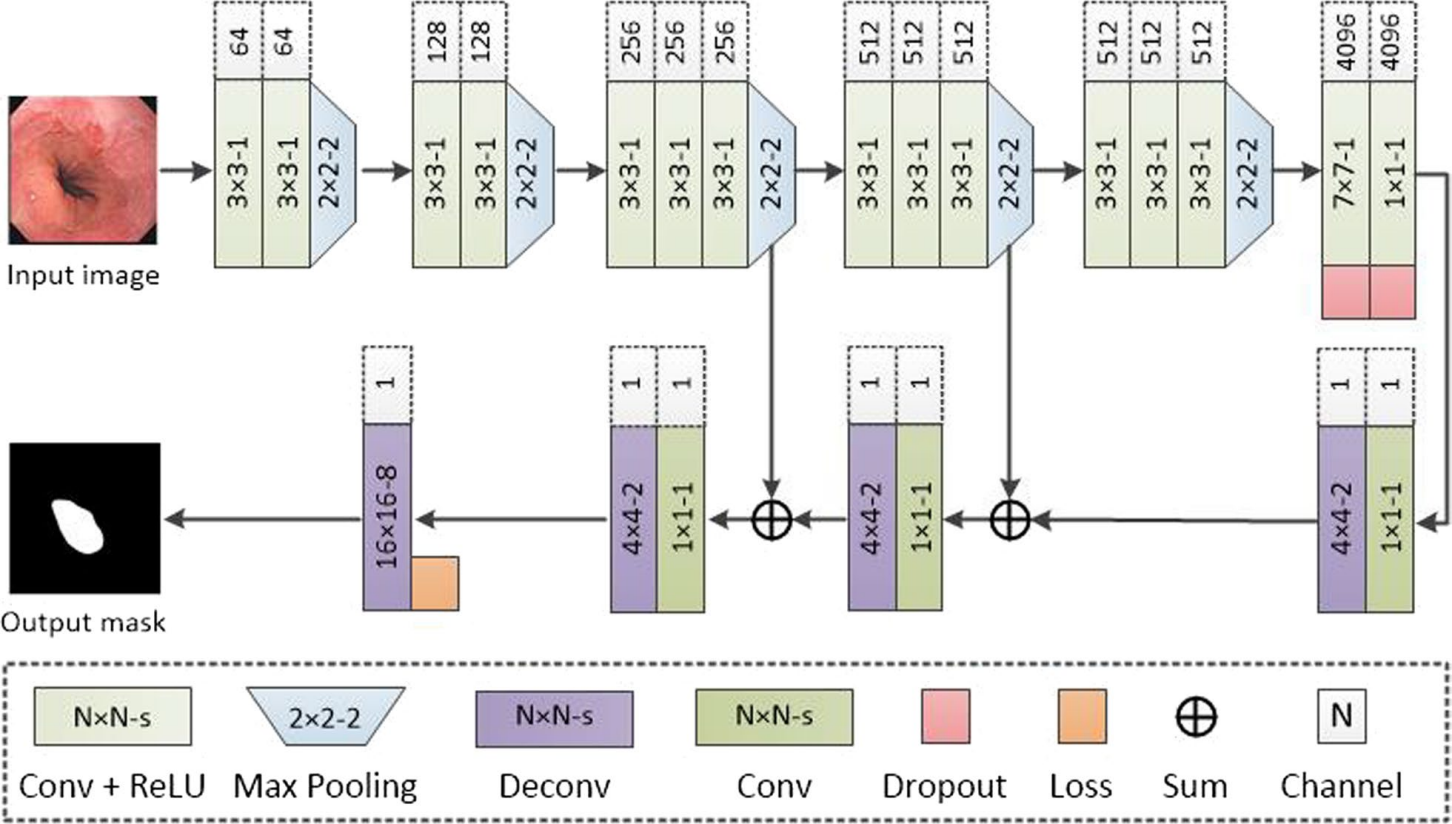
Schema of the FCN algorithm structure. Multiple full convolution layers with ReLU activation functions were used with deconvolution layers with skips. The images were input into the FCN and the segmentations were obtained as output masks in the same sizes

In this study, we developed a DL algorithm in a neural network structure of fully convolutional networks (FCN) [30]. As shown in Fig. 2, the neural network adopted several layers of fully convolutional neural network layers to extract abstract feature maps of an input image. After the downsampling, the deconvolutional neural network layers were appended to conduct the upsampling to generate the output image in the same size as the input image. Skip architectures were fused to both deep and shallow layers to achieve semantic segmentation at the pixel level. Furthermore, FCN is capable of processing images of any size, which allows FCN to be more suitable for medical images of various sizes. In the training stage, each image was input into the FCN, and a corresponding mask was generated to indicate the segmentation. The segmentation was compared against the ground truth obtained by experts. The loss formation was used to train the FCN. When all images in the training set were used to update the network, a trained FCN was obtained and passively used to generate segmentations for any inputs.

Since the BE scopes usually have two rims, namely GEJ and SCJ. We considered two approaches. Firstly, we trained two FCN networks independently to achieve the segmentation for GEJ and SCJ. In other words, two independent FCN networks were trained and evaluated using the annotations to obtain the rims of GEJ and SCJ. Secondly, we segmented the GEJ and SCJ using one single trained network. We reported and compared the performance of the two approaches. The obtained segmentations were further visualized for examinations.

To train and test the developed DL algorithm, we randomly divided the collected 443 images from 187 patients into two independent subsets according to patients. This approach ensured no images from a given individual patient appeared in both training and testing sets. In result, we obtained two subsets, namely a training set (n = 150, 354 images, 80%) and a test set (n = 37, 89 images, 20%) (Table 2). According to the Prague Classification, we divided the test set into 16 groups for analysis. The DL algorithm was first trained using the annotated images in the training set. Afterward, the trained DL algorithm was evaluated in the test set.

**Table 2:** Patients were randomly divided into one training set (80%) and one test set (20%) according to patients

Dataset	Patients	Images
Training set	150 (80%)	354
Test set	37 (20%)	89

The FCN neural network was implemented in the programming language of Python (3.7.3) using publicly available libraries of PyTorch (1.1.0), CUDA (10.1), and NumPy (1.16.2). The algorithm was trained and evaluated in a DL server equipped with a Tesla P40 graphic processing unit (GPU) running the operating system CentOS Linux (7.6.1810). Though a DL server was utilized in this study, it’s believed that a conventional workstation nowadays could be used to deploy the trained DL algorithm and generate segmentations within an acceptable time.

### Statistical analysis

In line with previous studies of image segmentation, the metric of intersection over union (IOU) was used to measure the performance of the DL algorithms. Intuitively, IOU indicated how well the predicted segmentation overlapped with the ground truth. A larger value of IOU closes to one indicates a favorable segmentation performance for a given algorithm. We also reported the Dice similarity coefficient (DSC), which is similar to IOU and widely appears in literature [31]. However, we used IOU as the major measurement for its simplicity and wide acceptance in literature. Therefore, the overall performance was reported as the averaged IOU and DSC in the test set.

## Results

### Performance

**Fig. 3:**
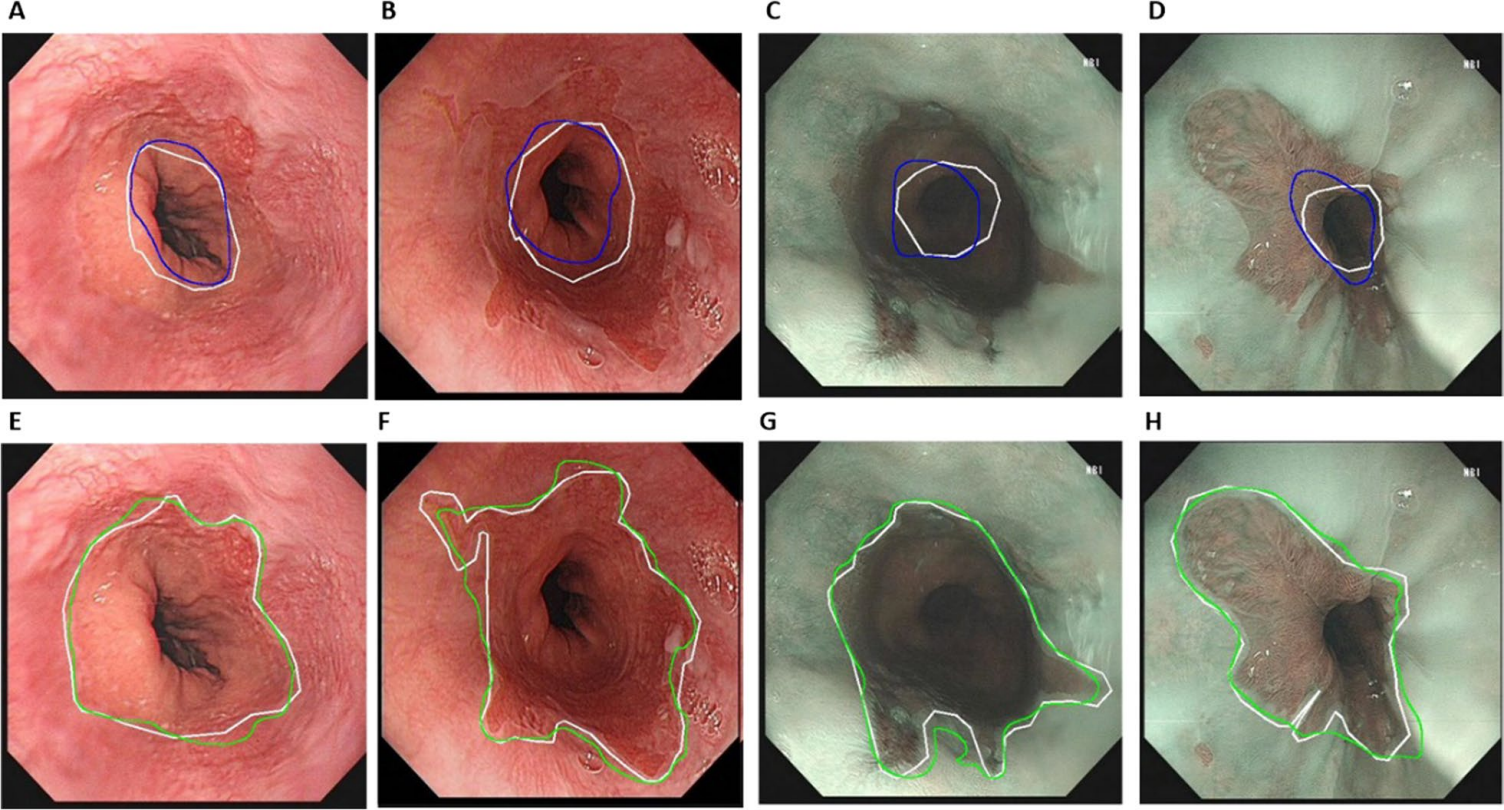
Examples of results obtained by DL algorithms versus expert annotations of four patients. Each column belongs to one patient. The upper row and lower row were GEJ and SCJ, respectively. The first two columns were taken using white light imaging, while the last two columns were taken using narrow band imaging. The expert annotations were marked as white. The DL obtained GEJ was marked as blue (upper row). The IOUs for GEJ were 0.79 (A), 0.76 (B), 0.66 (C), and 0.66 (D). The DL obtained SCJ was marked as green (lower row). The IOUs for SCJ were 0.91 (E), 0.88 (F), 0.91 (G), and 0.94 (H)

As described above, the DL algorithms were developed to obtain the GEJ and SCJ separately in two approaches. In Table 3, we reported the results of segmentation achieved by FCN for GEJ and SCJ in the test set. We found that the approach of separately segmenting GEJ and SCJ using two FCN networks was optimal than the approach of using one single network. To illustrate the results of DL in the identification of BE scopes, we visualized the segmentations of both DL and experts for representative samples in Fig. 3. As shown, DL was capable to accurately identify the GEJ and SCJ of BE scopes. Experts examined the DL results for all images in the test set and concluded that the agreement between DL results and expert annotations was satisfying. For those cases with smaller values of IOU, the overall shapes of GEJ and SCJ obtained by DL were also acceptable. By investigating the average IOU values for each subset and the whole set, we found that there is no significant difference among subsets and the whole set. In the subsets, average IOU values ranged from 0.32 to 0.68 for the GEJ and from 0.60 to 0.94 for the SCJ, respectively.

**Table 3:** Performance of DL algorithm achieved in the test set in the tasks of identifying the GEJ and the SCJ of the BE scopes

GEJ/SCJ	IOU		DSC	
	Average	SD	Average	SD
GEJ	0.56	0.14	0.71	0.12
SCJ	0.82	0.12	0.90	0.08
GEJ + SCJ	0.66	0.13	0.79	0.11

## Discussion

EAC is the main histological type of esophageal cancer in the west [32], and BE is the only known histological precursor of EAC. Recently, several reports indicated that the incidences of BE and Barrett’s esophageal adenocarcinoma (BEA) were rising in Asia [33, 34]. Previous studies have shown that prior diagnosis, surveillance [7, 35–37], and appropriate treatment practices [38, 39] of BE can reduce the risk of EAC progression and improve survival. Endoscopic biopsy is the most commonly used method for diagnosis and monitoring of BE [40], and endotherapy such as endoscopic resection and esophageal ablation becomes the standard of care for BE [41]. These measures all require endoscopists to accurately identify the scopes of BE under endoscopic examination [42, 43]. This process relies on the experience of individuals with inevitable misjudgments, variations, and time consumption. Moreover, the diversities in shapes, appearances, and textures of BE contribute to the difficulties of accurate segmentation of BE scopes.

Therefore, in this study, we proposed and developed a DL method to automatically segment the BE scopes in endoscopy, which could further improve early-accurate diagnosis and treatments of BE. We collected 443 images from 187 patients and invited experts to manually annotate BE scopes. We constructed one training set and one test set to develop and evaluate the DL methods.

Mendel et al. included 100 endoscopic images, including 50 cancer cases and 50 BE cases of 39 patients [20]. Using a migration-based learning approach, they reported a sensitivity of 0.94 and specificity of 0.88. However, the BE cases were relatively insufficient. Wu et al. developed neural networks to segment 797 endoscopic images of cancer, BE, and inflammation cases [21]. Sharib et al. used a depth estimator network to measure C&M values in 194 high-definition videos from 131 BE patients [27]. Compared to the above works, our study dedicated to the automated identification and location of BE scopes in endoscopic images using DL. Additionally, we developed DL methods to accurately identify both GEJ and SCJ. It is worth mentioning that our approach of separately segment GEJ and SCJ using two DL networks outperformed the traditional approach using one single DL network was inspiring for similar medical image analysis tasks. These efforts could assist endoscopists in the diagnosis of BE efficiently and improve the accuracy of diagnosis of BE.

However, there are still several limitations in this study. Firstly, we only focused on developing DL to automated segment the scopes of BE under esophagoscopic examination but did not differentiate from other esophageal lesions. In the future, we would include other esophageal lesions and extend the present DL framework to classify and diagnose types of BE. Secondly, this is a retrospective study from a single center. The results could be further validated in prospective studies using external cohorts. Thirdly, the developed DL methods in this study are still in rapid evolution with more emerging advanced DL algorithms. It’s expectable to evaluate new DL algorithms to diagnose BE in endoscopic images to further improve the performance. Current guidelines recommend that the diagnosis of BE should be based on the presence of SCJ of 1 cm proximal to the EGJ, with biopsy results consistent with those of intestinal [44–46]. AI could accurately quantify the elevation of SCJ and objectively evaluate BE, thereby avoiding over-diagnosis and over-follow-up. The application of AI to exclude the elevation of SCJ more than 1 cm is worth further investigation.

In this study, we carried out the recognition and segmentation of the BE scope in endoscopic images using DL. Specifically, FCN neural networks were developed and evaluated. The DL methods achieved satisfying performance in the segmentation of GEJ and SCJ, indicating their promising potentials in clinical BE evaluations.

## Data Availability

The datasets during and/or analyzed during the current study available from the corresponding author on reasonable request.

## Abbreviations

BE: Barrett's esophagus
EAC: esophageal adenocarcinoma
ESO: esophagoscopy
GEJ: gastroesophageal junction
SCJ: squamous-columnar junction
AI: artificial intelligence
DL: deep learning
FCN: fully convolutional networks
CNN: convolutional neural network
IOU: intersection over union
DSC: Dice similarity coefficient
BEA: Barrett’s esophageal adenocarcinoma
